# Acute recanalization therapy for ischemic stroke during pregnancy and puerperium

**DOI:** 10.1007/s00415-024-12313-4

**Published:** 2024-04-03

**Authors:** Anna Richardt, Karoliina Aarnio, Aino Korhonen, Kirsi Rantanen, Liisa Verho, Sami Curtze, Hannele Laivuori, Mika Gissler, Minna Tikkanen, Petra Ijäs

**Affiliations:** 1 Neurology, University of Helsinki and Helsinki University Hospital, Helsinki, Finland; 2 Obstetrics and Gynecology, University of Helsinki and Helsinki University Hospital, Helsinki, Finland; 3 Medical and Clinical Genetics, University of Helsinki and Helsinki University Hospital, Helsinki, Finland; 4 Institute for Molecular Medicine Finland, Helsinki Institute of Life Science, University of Helsinki, Helsinki, Finland; 5 Department of Obstetrics and Gynecology, Tampere University Hospital, The Wellbeing Services County of Pirkanmaa, Finland; 6 Center for Child, Adolescent and Maternal Health Research, Faculty of Medicine and Health Technology, Tampere University, Tampere, Finland; 7 Department of Knowledge Brokers, Finnish Institute for Health and Welfare, Helsinki, Finland; 8 Academic Primary Health Care Centre, Region Stockholm, Stockholm, Sweden; 9 Obstetrics and Gynecology, University of Helsinki, Helsinki, Finland; 10 Department of Molecular Medicine and Surgery, Karolinska Institute, Stockholm, Sweden

**Keywords:** Ischemic stroke, Pregnancy, Puerperium, Recanalization therapy, Intravenous thrombolysis, Endovascular thrombectomy

## Abstract

**Background:**

The safety and efficacy of intravenous thrombolysis (IVT) and endovascular thrombectomy for an ischemic stroke (IS) during pregnancy and puerperium are poorly studied. We evaluated the complications and outcome of recanalization therapy in maternal ISs.

**Methods:**

A nationwide cohort of maternal ISs in Finland during 1987–2016 was collected by linking national healthcare registers: Medical Birth Register, Hospital Discharge Register, and Cause-Of-Death Register. The diagnoses were verified retrospectively from patient records. IVT-treated patients were compared to controls, who were young females with non-pregnancy-related IS from the Helsinki Stroke Thrombolysis Registry.

**Results:**

Totally, 12 of 97 (12.4%) maternal ISs were treated with recanalization therapy. Compared to controls, IVT-treated maternal IS patients had more frequently early (age-adjusted odds ratio (aOR) = 7.63, 95% CI 1.49–39.04) and major (aOR = 8.59, 95% CI 2.09–35.31) neurological improvements, measured using the National Institute of Health Stroke Scale. Good functional outcomes (modified Rankin Scale 0–2) at three months were equally common in maternal ISs and controls. No other complications were observed in IVT-treated maternal ISs than 1 (9.1%) symptomatic nonfatal intracranial hemorrhage. Among maternal IS patients treated with recanalization or conventional therapy, good functional outcome at the end of the follow-up was less common in recanalization-treated patients (66.7% vs 89.4%, aOR = 0.22, 95% CI 0.052–0.90), but otherwise outcomes were similar.

**Conclusions:**

In this small nationwide cohort of maternal ISs, the complications of recanalization therapy were rare, and the outcomes were similar in IVT-treated maternal IS patients and controls. Maternal ISs should not be excluded from recanalization therapy in otherwise eligible situations.

## Introduction

Ischemic stroke (IS) during pregnancy and puerperium, i.e. maternal IS, is a rare but devastating cause of maternal disability and mortality, with an estimated incidence of 5.5 per 100,000 deliveries [1, 2]. Intravenous thrombolysis (IVT) and endovascular thrombectomy (EVT), i.e., recanalization therapies, are the standard treatment of acute IS [3]. The safety and efficacy of recanalization therapies during pregnancy and the postpartum period are poorly known. Pregnant and postpartum patients have been excluded from randomized controlled trials on acute recanalization treatments, and most publications are either case reports or small series [4, 5].

Few retrospective register-based case–control studies on IVT or EVT during pregnancy or the puerperium have been performed [6–8]. The short-term outcome and the risk of complications seem to be similar in pregnant, postpartum, and nonpregnant IS patients treated with recanalization therapy [6–8]. According to the American Stroke Association guidelines, IVT can be considered as a treatment for pregnant women with a moderate to severe acute IS if the predicted benefits outweigh the risks [3, 4]. The Canadian Stroke Best Practice Consensus recommends considering IVT with alteplase in a disabling stroke, and EVT in proximal large vessel occlusion during pregnancy [9]. While the European Stroke Organization (ESO) does not provide recommendations on recanalization therapies during pregnancy and puerperium due to insufficient evidence, it suggests that IVT during pregnancy can be considered on an individual basis, and EVT can be used [5]. The ESO urged that more studies are required to evaluate the safety and efficacy of IVT and EVT in acute IS during pregnancy and the puerperium.

We collected a nationwide cohort of IS patients treated with IVT or EVT during pregnancy or the puerperium in Finland from 1987 to 2016. In this study, we examined the outcomes and complications of recanalization therapy in women with IS during pregnancy and the puerperium. The objectives are to identify if the outcomes and complications are similar in IVT-treated pregnant or postpartum women compared to women of the same age group with IS not related to pregnancy. We also assessed the differences in clinical features and outcomes in pregnant or postpartum IS patients receiving recanalization therapy compared to those not receiving such treatment to evaluate the possibility that eligible women were excluded from recanalization therapy due to pregnancy.

## Methods

This article follows the STrengthening the Reporting of OBservational studies in Epidemiology (STROBE) reporting guideline (Online Resource 1) [10].

### Study design and identification of the patients from the national registers

We performed a retrospective, nationwide study of ISs associated with pregnancy and the postpartum period in Finland, as well as a nested case–control study. Women diagnosed with IS during pregnancy or the postpartum period in Finland between 1987 and 2016 were identified utilizing national healthcare registers: Medical Birth Register (MBR) [11], Hospital Discharge Register, i.e., Care Register for Health Care (HDR) [12], and the Cause of Death Register. The HDR includes inpatient hospitalizations since 1969 and outpatient visits in public hospitals since 1998 in Finland. The MBR includes all deliveries after the gestational age of ≥ 22 + 0 weeks or with a birth weight of ≥ 500 g.

The search strategy was comprehensive to find all cases. The International Statistical Classification of Diseases and Related Health Problems (ICD) and NOMESCO Classification of Surgical Procedures (NCSP) codes indicating a stroke or its treatment in the HDR, from nine months before delivery to three months after the delivery date in the MBR, were used to identify the patients. The diagnostic codes used to identify patients with IS during pregnancy or the puerperium were 4330A, 4331A, 4339A, 4340A, 4341A, 4349A in ICD-9 and I63 in ICD-10, and the procedure codes used were AAL10, TPX22, PA2AT, PA2BT, PA2CT, PA2ST, PA2VT, PAE12, PAE14, PAF12, PAF13, PAF14, PAF15, PA2YT, PA6YT, PA7XT, PA7YT, PA8YT, PAQ12, PAQ14, PA4AT, PA4YT, PA6AT, PAF12, and PAF14. The causes of death were acquired from the Cause of Death Register for all cases identified from the HDR. Additionally, the Cause of Death Register was searched for maternal deaths due to a stroke with codes 6740A and O99.4 during the years 1987–2016. Perinatal mortality was collected from the MBR, and it was defined as a death from 22 weeks of gestation until the age of 1 week.

### Chart verification and definitions

Medical records were acquired from healthcare facilities where the disease or procedure codes indicating IS during pregnancy or puerperium were coded. The charts were obtained until the end of the year 2016 in specialties of neurology, neurosurgery, gynecology, internal medicine, radiology, and laboratory medicine. Clinical data were collected from the medical records. The diagnosis of IS, its temporal connection to pregnancy, demographics, treatment, complications, and outcomes were verified from the medical records by stroke neurologists.

Patients who did not have IS during pregnancy or the puerperium were excluded, and the reasons of exclusion were recorded. Only the IS cases of arterial origin were included. The definition of IS was brain cell death attributable to ischemia, based on the pathological, imaging, or other objective evidence of a cerebral focal ischemic injury in a defined vascular distribution; or clinical evidence of a cerebral focal ischemic injury based on symptoms persisting ≥ 24 h or until death, and other etiologies excluded [13]. Pregnancy was defined as an event starting at conception and the postpartum period lasting until 12 weeks after the delivery. Recanalization therapy includes IVT and EVT. IVT was defined as receiving intravenous thrombolytic medication, i.e., alteplase as 0.9 mg/kg 10% bolus and 60 min of infusion. EVT was defined as mechanical thrombectomy and/or intra-arterial administration of thrombolytic medications.

The severity of IS was evaluated based on the National Institutes of Health Stroke Scale (NIHSS) score [14]. Early neurological improvements were defined as the NIHSS score improving by ≥ 4 points and major neurological improvements as the NIHSS score improving by ≥ 8 points from the baseline to 24 h after treatment. The level of consciousness at hospital arrival was measured with the Glasgow Coma Scale (GCS) [15]. The functional outcome was measured based on the Modified Rankin Scale (mRS) [16]. A good functional outcome was defined as a mRS score of 0–2. The NIHSS, GCS, and mRS scores were estimated for maternal IS patients by our research group’s neurologist using patient records if the scores were not recorded by clinicians during the initial visit. The NIHSS and mRS scores for the Helsinki Stroke Thrombolysis registry’s controls were recorded by clinicians during the hospital admission and at the three-month control visit.

Intracerebral hemorrhages (ICHs) were classified based on the European Cooperative Acute Stroke Study (ECASS) criteria for hemorrhagic infarction (HI) grades 1 and 2, and parenchymal hemorrhage (PH) grades 1 and 2 [17]. Per the criteria, HI grade 1 was small petechiae along the margins of the infarct, and HI grade 2 was confluent petechiae within the infarcted area without a space-occupying effect. Moreover, PH grade 1 was a clot less than 30% of the infarcted area with a mild space-occupying effect, and PH grade 2 was a dense blood clot exceeding 30% of the infarcted area with a significant space-occupying effect. Symptomatic ICH was defined, according to the National Institute of Neurological Disorders and Stroke (NINDS) criteria, as a hemorrhage causing any decline in the patient’s neurological status [18]; and according to the ECASS III criteria, as a hemorrhage causing neurological deterioration, indicated by an NIHSS score increasing by ≥ 4 points, or causing death [19]. Computer tomography (CT) scans were taken prior to treatment, at 24 ± 6 h after treatment, and otherwise if necessary. Using The Oxfordshire Community Stroke Project (OCSP) classification, ISs were classified into total anterior circulation, partial anterior circulation, lacunar, and posterior circulation infarcts [20]. The etiologies of ISs were defined using the Trial of ORG 10172 in Acute Stroke Treatment (TOAST) criteria, which included large-artery atherosclerosis, cardioembolism, small-vessel occlusion, stroke of other determined etiology, and stroke of undetermined etiology [21].

### Case–control analysis

Controls were obtained from Helsinki Stroke Thrombolysis Registry and matched by age distribution and sex: 18–50-year-old women who were diagnosed with acute IS not related to pregnancy and received IVT in Helsinki University Hospital during the years 1999–2022. Patients who were pregnant or up to 12 weeks postpartum at the stroke onset (n = 4) and patients with a clear non-ischemic cause for their symptoms (i.e., demyelination, tumor) (n = 6) based on patient records were excluded, resulting in a total of 197 controls. We compared them to pregnant or postpartum IS patients who received IVT (n = 11) from 1987 to 2016 in Finland. These groups were compared by demographics, clinical features at hospital arrival, the time to treatment, hemorrhagic complications, mortality, and functional outcomes.

In sensitivity analyses, we compared women diagnosed with IS during pregnancy or the puerperium in Finland from 1987 to 2016 and received IVT and/or EVT (n = 12) to those who were not treated with recanalization therapy (n = 85). These groups were compared by demographics, medical histories, stroke features, complications, mortalities, functional outcomes, the length of their hospital treatments, and discharge destinations.

### Outcomes

The primary outcomes were functional outcomes at the three-month follow-up after the recanalization treatment and hemorrhagic complications. The secondary outcomes were neurological improvements (measured by a NIHSS score improvement by ≥ 4 or ≥ 8 points), the length of hospital stays, as well as maternal and perinatal mortalities.

### Statistical methods

Data were presented as numbers and percentages for categorical variables, and as the mean and standard deviation or median and interquartile range (IQR) for continuous variables. A binary logistic regression model was used to analyze the unadjusted and age-adjusted odds ratios (ORs) and 95% confidence intervals (CI).

## Results

### Maternal IS cohort

**Fig. 1:**
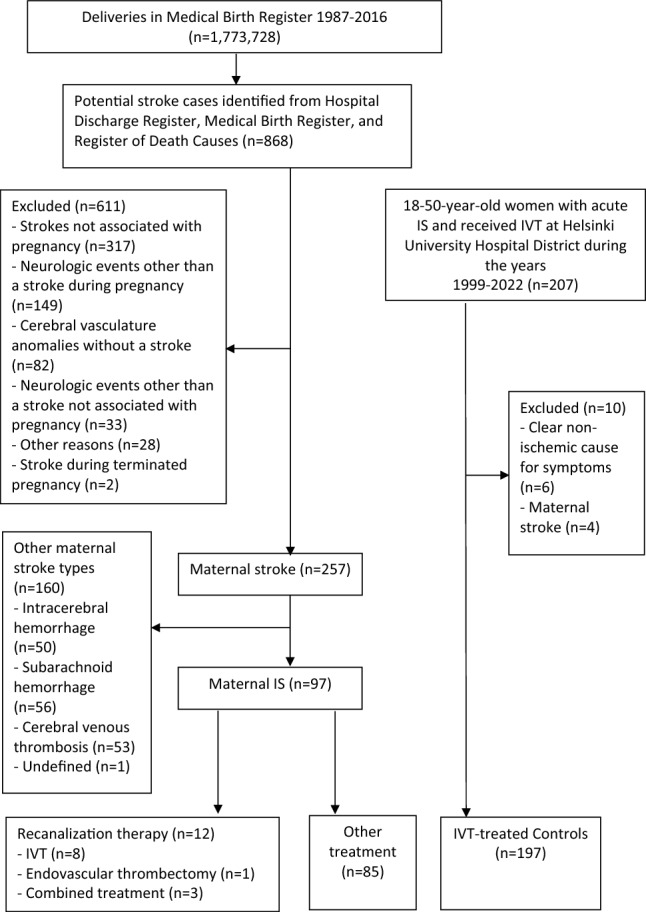
Flowchart of the maternal IS cohort and controls. *IS* ischemic stroke, *IVT* intravenous thrombolysis

During the years 1987–2016, there were 97 pregnant or postpartum IS patients, of whom 12 (12.4%) received recanalization therapy in Finland (Fig. 1). In total, eight patients were treated with IVT, one patient solely with EVT and three patients with combination therapy. Six patients were treated with recanalization therapy during pregnancy (5 IVT and 1 EVT) and six patients during the postpartum period (3 IVT and 3 combination therapy). In pregnant patients, the median gestational age was 19 weeks (IQR 11–22) when receiving any form of recanalization therapy and 16 weeks (IQR 11–23) when receiving IVT, both with a range from 10 weeks and 4 days to 23 weeks and 1 day. During the postpartum period, the treatment was administered between 10 and 84 days after delivery (median: 24, IQR: 10–56). Of the patients treated with recanalization therapy, 6 (50.0%) had brain CT, 6 (50.0%) had brain magnetic resonance imaging (MRI) and 11 (91.7%) had vessel imaging (computed tomography angiography, magnetic resonance angiography or digital subtraction angiography) before administration of the treatment.

### Case–control analysis of patients receiving IVT

A total of 11 patients with IS during pregnancy or the postpartum period and 197 controls with IS not related to pregnancy, who all received IVT, were included in the case–control analysis (Table 1). Maternal IS patients were younger with a median age of 30 years (IQR: 28–41) compared to the controls with a median age of 43 years (IQR: 34–47) (unadjusted OR = 0.93, 95% CI 0.87–0.99). Compared to controls, maternal IS patients had a higher percentage of dyslipidemia (36.4% vs 13.2%, age-adjusted odds ratio (aOR) = 8.47, 95% CI 1.82–39.52) and a history of IS (18.2% vs 3.6%, aOR = 5.43, 95% CI 1.01–11.51), but other baseline characteristics did not differ. Wake-up strokes were more common among maternal IS patients than in controls (27.3% vs 5.1%, aOR = 13.87, 95% CI 2.56–75.13). The median symptom-to-needle time was 110 min in cases and 123 min in controls (aOR = 1.01, 95% CI 0.99–1.03). In wake-up strokes, the median time between the last-seen-well and treatment onset was 305 min (IQR 166–463).

**Table 1:** Comparison of IVT-treated maternal and other IS patients

	All	Maternal IS	Controls	Age-adjusted OR (95% CI)
No. of cases (%)	208 (100.0)	11 (5.3)	197 (94.7)	
Clinical features				
Age (years)	42.0 (33–46)	30.0 (28–41)	43.0 (34–47)	
Smoking^a^	53 (32.1)	3 (27.3)	50 (32.5)	0.62 (0.15–2.57)
Hypertension	36 (17.3)	0	36 (18.3)	N/A
Diabetes	4 (1.9)	0	4 (2.0)	N/A
Dyslipidemia	30 (14.4)	4 (36.4)	26 (13.2)	8.47 (1.82–39.52)*
History of IS	9 (4.3)	2 (18.2)	7 (3.6)	5.43 (1.01–11.51)*
Cardiac disease	10 (4.8)	0	10 (5.1)	N/A
Medications before admission				
Antihypertensive	31 (14.9)	0	31 (15.7)	N/A
Antithrombotic	15 (7.2)	1 (9.1)	14 (7.1)	1.86 (0.21–16.71)
Anticoagulation	2 (1.0)	0	2 (1.0)	N/A
Statin	14 (6.7)	0	14 (7.1)	N/A
Stroke onset				
Wake-up stroke	13 (6.3)	3 (27.3)	10 (5.1)	13.87 (2.56–75.13)*
Systolic BP, mmHg^b^	137 (126–151)	135 (116–147)	139 (126–151)	0.99 (0.95–1.02)
Diastolic BP, mmHg^b^	82 (73–92)	74 (71–85)	83 (74–92)	0.96 (0.91–1.02)
Glucose, mmol/l	5.6 (5.0–6.0)	4.9 (4.6–5.6)	5.9 (5.0–6.2)	0.58 (0.28–1.23)
NIHSS before treatment	6 (3–10)	10 (5–11)	5 (3–10)	1.06 (0.97–1.15)
Moderate or severe stroke (NIHSS ≥ 5)	126 (60.6)	9 (81.8)	117 (59.4)	3.24 (0.67–15.64)
Intra-arterial procedure done	31 (14.9)	3 (27.3)	28 (14.2)	2.56 (0.61–10.70)
Symptom-to-needle time (min)^c^	122 (84–175)	110 (83–161)	123 (82–174)	0.99 (0.98–1.01)
Door-to-needle time (min)^d^	27 (17–51)	33 (20–55)	27 (16–51)	1.01 (0.99–1.03)
Radiographic features				
Early infarct signs	44 (21.2)	3 (27.3)	41 (20.8)	1.53 (0.38–6.18)
Hyperdense artery sign	70 (33.7)	7 (63.6)	63 (32.0)	3.36 (0.93–12.12)
Infarct visible at 24h	125 (60.1)	9 (81.8)	116 (58.9)	2.64 (0.55–12.8)
Etiology of IS by TOAST classification				
Large artery atherosclerosis	6 (2.9)	0	6 (3.0)	N/A
Cardioembolism	41 (19.7)	3 (27.3)	38 (19.3)	1.01 (0.24–4.17)
Small vessel disease	5 (2.4)	0	5 (2.5)	N/A
Other defined	45 (21.6)	2 (18.2)	43 (21.8)	0.50 (0.10–2.45)
Undetermined	56 (26.9)	6 (54.5)	50 (25.4)	2.23 (0.63–7.92)
Complications^e^				
Any ICH	14 (6.8)	2 (18.2)	12 (6.1)	4.97 (0.88–28.15)
HI1	6 (2.9)	1 (9.1)	5 (2.5)	7.00 (0.67–73.58)
HI2	4 (1.9)	0	4 (2.3)	N/A
PH1	1 (0.5)	0	1 (0.5)	N/A
PH2	3 (1.4)	1 (9.1)	2 (1.0)	6.74 (0.55–82.40)
Symptomatic ICH				
NINDS criteria	4 (1.9)	1 (9.1)	3 (1.5)	5.52 (0.50–60.74)
ECASS III criteria	2 (1.0)	0	2 (1.0)	N/A
Fatal symptomatic ICH	1 (0.5)	0	1 (0.5)	N/A
Outcomes				
NIHSS at 24 hours^f^	1 (0–3)	1 (0–3)	1 (0–3)	1.06 (0.89–1.27)
Early neurological improvement (NIHSS improves by ≥ 4 points)^f^	96 (47.8)	9 (81.8)	87 (45.8)	7.63 (1.49–39.04)*
Major neurological improvement (NIHSS improves by ≥ 8 points)^f^	45 (22.4)	6 (54.5)	39 (20.5)	8.59 (2.09–35.31)*
NIHSS score at 24 h 0–1^h^	117 (58.2)	8 (72.7)	109 (57.4)	2.35 (0.58–9.47)
In-hospital mortality^g^	1 (0.5)	0	1 (0.5)	N/A
Mortality at 3 months	3 (1.4)	0	3 (1.5)	N/A
Functional outcome (mRS) at 3 months^g^	1 (0–2)	2 (0–3)	1 (0–2)	1.14 (0.74–1.74)
Good functional outcome (mRS 0–2) at 3 months^g^	177 (85.9)	8 (81.8)	169 (86.7)	0.49 (0.10–1.65)

Data are presented as n (%) or median (interquartile range).

*BP* blood pressure, *CI* confidence interval, *HI* hemorrhagic infarction, *ICH* intracerebral hemorrhage, *IS* ischemic stroke, *mRS* Modified Rankin Scale, *N/A* not available, *OR* odds ratio, *PH* parenchymal hemorrhage, *TOAST* Trial of ORG 10172 in Acute Stroke Treatment. ORs and 95% CIs were calculated via logistic regression

*Indicates statistical significance

^a^43 controls missing

^b^1 case and 3 controls missing

^c^3 cases and 10 controls with a wake-up stroke missing

^d^2 cases missing

^e^1 control missing

^f^7 controls missing

^g^2 controls missing

The rate of any ICH was 18.2% (n = 2) in maternal IS patients and 6.1% (n = 12) in controls (aOR = 4.97, 95% CI 0.88–28.15). The occurrence of symptomatic ICH was 1 (9.1%) in maternal IS and 3 (1.5%) in controls according to the NINDS criteria (aOR = 5.52, 95% CI 0.50–60.74), and 0% in maternal IS and 2 (1.0%) in controls according to ECASS III criteria. At three months, none of the IVT-treated maternal IS patients had died, and the mortality rate was 1.5% in controls. There were no other complications except ICH among maternal IS patients during the median follow-up of 2.1 years (IQR 0.6–5.3). All maternal IS patients who received IVT delivered full-term babies, and there were no perinatal deaths.

Maternal IS patients had a higher occurrence of early neurological improvement (NIHSS score improvement by ≥ 4 points) than the controls (81.8% vs 45.8%, aOR = 7.63, 95% CI 1.49–39.04). Furthermore, 54.5% of maternal IS patients had a major neurological improvement (NIHSS score improvement by ≥ 8 points) compared to 20.5% of the controls (aOR = 8.59, 95% CI 2.09–35.31). The median mRS score at three months was 2 (IQR 0–3) in maternal IS patients and 1 (IQR: 0–2) in the controls (aOR = 1.14, 95% CI 0.74–1.74). The proportion of patients having a good functional outcome (mRS: 0–2) at three months was similar in both groups (81.8% of maternal IS patients vs 86.7% of controls, aOR = 0.49, 95% CI 0.10–1.65).

### Comparison of maternal IS patients receiving recanalization therapy versus other treatment

Of 97 patients diagnosed with IS during pregnancy or the postpartum period in Finland between 1987 and 2016, 12 patients (12.4%) received recanalization therapy and 85 patients (87.6%) received another treatment than recanalization therapy. The median age of IS patients was 30 for both groups (unadjusted OR = 1.09, 95% CI 0.97–1.22). Patients who underwent recanalization therapy did not have hypertensive disorders of pregnancy, while 32.9% of patients receiving other treatments had them. The primary admission site was the stroke center in 75.0% of patients who received recanalization therapy compared to 25.9% of patients receiving other treatments (aOR = 8.10, 95% CI 1.98–33.07). In the recanalization therapy group, partial anterior strokes were more common (75.0 vs 35.3%, aOR = 5.27, 95% CI 1.31–21.20) and posterior strokes less common (8.3% vs 47.1%, aOR = 0.12, 95% CI 0.014–0.91) compared to those who received other treatments. The etiology of IS by TOAST classification was more frequently cardioembolism in patients treated with recanalization therapy compared with patients receiving other treatments (33.3% vs 10.6%, aOR = 4.72, 95% CI 1.11–19.99). Other baseline characteristics were similar.

The occurrence of hemorrhagic transformation causing symptoms was similar between patients who received recanalization treatment or other treatment (8.3% vs 3.5%, aOR = 2.49, 95% CI = 0.24–26.03, by the NINDS criteria, and 0% vs 1.2%, by the ECASS III criteria). The maternal in-hospital mortality was 8.3% in patients with recanalization therapy and 4.7% in patients with other treatments (aOR = 1.61, 95% CI 0.16–16.25). There were no additional deaths until the end of the follow-up time (median: 5.9 years, IQR 1.6–16.6). Among the patients who received recanalization therapy, the only deceased patient had solely EVT during pregnancy, and the cause of death was endocarditis 1 month from stroke onset. All patients who had recanalization therapy during pregnancy delivered live babies, four through a vaginal delivery and two through a cesarean section. The median length of their hospital stay was similar, eight days in patients treated with recanalization and other treatments (aOR = 1.01, 95% CI 0.95–1.07). A good functional outcome (mRS: 0–2) at the end of the follow-up period was less common if the patient had received recanalization treatment compared to other treatments (66.7% vs 89.4%, aOR = 0.22, 95% CI 0.052–0.90). The median follow-up time was shorter in patients with recanalization therapy than patients with other treatments (2.0 vs 7.6 years, aOR = 0.85, 95% CI 0.74–0.98) (Table 2).

**Table 2:** Comparison of pregnant and postpartum ischemic stroke patients with recanalization therapy versus other treatment

	All	Recanalization therapy	Other treatment	Age-adjusted OR (95% CI)
No. of cases (%)	97 (100.0)	12 (12.4)	85 (87.6)	
Clinical features				
Age (years)	30 (26–35)	30 (28–40)	30 (26–34)	
Parity	1 (0–2)	1 (0–3)	1 (0–2)	1.12 (0.88–1.42)
BMI, kg/m^2a^	23 (21–27)	23 (22–28)	23 (21–26)	1.00 (0.88–1.15)
Pregnant at stroke onset	43 (44.3)	6 (50.0)	37 (43.5)	1.45 (0.42–5.00)
Smoking	19 (19.6)	3 (25.0)	16 (18.8)	1.49 (0.35–6.30)
Hypertension	7 (7.2)	0	7 (8.2)	N/A
Diabetes mellitus	0	0	0	N/A
Dyslipidemia	35 (36.1)	4 (33.3)	31 (36.5)	0.74 (0.20–2.77)
Cardiac disease	9 (9.3)	2 (16.7)	7 (8.2)	3.21 (0.53–19.46)
Prothrombotic disease	3 (3.1)	0	3 (3.5)	N/A
Gestational diabetes	16 (16.5)	2 (16.7)	14 (16.5)	0.96 (0.18–4.96)
Pre-eclampsia or eclampsia	16 (16.5)	0	16 (18.8)	N/A
Hypertensive disorders of pregnancy^b^	28 (28.9)	0	28 (32.9)	N/A
Caesarean section	26 (26.8)	3 (25.0)	23 (27.1)	0.88 (0.21–3.59)
In Vitro Fertilization	4 (4.1)	0	4 (4.7)	N/A
Gestational age at stroke onset				
1st trimester (≤ 12 + 0 weeks)	14 (14.4)	2 (16.7)	12 (14.1)	1.13 (0.97–1.33)
2nd trimester (12 + 1 to 28 + 0 weeks)	19 (19.6)	4 (33.3)	15 (17.6)	1.15 (0.98–1.36)
3rd trimester (≥ 28 + 1 weeks)	10 (10.3)	0	10 (11.8)	N/A
Postpartum	54 (55.7)	6 (50.0)	48 (56.5)	0.69 (0.20–2.37)
Stroke onset				
Primary admission site stroke center	31 (32.0)	9 (75.0)	22 (25.9)	8.10 (1.98–33.07)*
GCS at arrival^c^	15 (15–15)	15 (15–15)	15 (15–15)	N/A
Systolic BP^a^	133 (120–150)	135 (115–145)	133 (123–150)	1.03 (0.99–1.06)
Diastolic BP^a^	80 (71–91)	72 (71–84)	82 (75–94)	0.92 (0.86–0.99)*
Stroke location				
Total anterior	10 (10.3)	2 (16.7)	8 (9.4)	2.52 (0.44–14.41)
Partial anterior	40 (41.2)	9 (75.0)	30 (35.3)	5.27 (1.31–21.20)*
Lacunar stroke	6 (6.2)	0	6 (7.1)	N/A
Posterior stroke	41 (42.3)	1 (8.3)	40 (47.1)	0.12 (0.014–0.91)*
Infarct visible in neuroimaging at 24h^d^	59 (78.7)	10 (83.3)	49 (77.8)	1.42 (0.27–7.30)
Etiology of IS by TOAST classification				
Large artery atherosclerosis	0	0	0	N/A
Cardioembolism	13 (13.4)	4 (33.3)	9 (10.6)	4.72 (1.11–19.91)*
Small vessel disease	2 (2.1)	0	2 (2.4)	N/A
Other defined	27 (27.8)	2 (16.7)	25 (29.5)	0.38 (0.075–1.95)
Undetermined	55 (56.7)	6 (50.0)	49 (57.6)	0.87 (0.25–3.06)
Outcomes and complications				
Hemorrhagic transformation				
Any ICH	11 (11.3)	3 (25.0)	8 (9.4)	3.16 (0.68–14.69)
Symptomatic ICH	1 (1.0)	0	1 (1.2)	N/A
NINDS criteria	4 (4.1)	1 (8.3)	3 (3.5)	2.49 (0.24–26.03)
ECASS III criteria	1 (1.0)	0	1 (1.2)	N/A
Fatal ICH	0	0	0	N/A
Term delivery	74 (76.3)	11 (91.7)	63 (74.1)	4.12 (0.50–34.22)
Perinatal mortality	3 (3.1)	0	3 (3.5)	N/A
Maternal in-hospital mortality	5 (5.2)	1 (8.3)	4 (4.7)	1.61 (0.16–16.25)
Follow-up time (years)	5.9 (1.6–16.6)	2.0 (0.5–5.1)	7.6 (2.0–18.0)	0.85 (0.74–0.98)*
Functional outcome (mRS)				
At discharge	2 (1–3)	2 (1–3)	2 (1–3)	1.29 (0.89–1.87)
At 3 months	1 (0–2)	2 (0–3)	1 (0–2)	1.27 (0.90–1.79)
End of follow up	1 (0–2)	2 (0–3)	1 (0–1)	1.31 (0.94–1.83)
Good functional outcome (mRS 0–2)				
At discharge	71 (73.2)	7 (58.3)	64 (75.3)	0.45 (0.13–1.60)
At 3 months	81 (83.5)	8 (66.7)	73 (85.9)	0.32 (0.081–1.26)
End of follow-up	83 (87.4)	8 (66.7)	76 (89.4)	0.22 (0.052–0.90)*
Treatment at intensive care unit	14 (14.4)	1 (8.3)	13 (15.3)	0.42 (0.048–3.58)
Discharge to home	80 (82.5)	10 (83.3)	70 (82.4)	1.03 (0.20–5.27)
Hospital length of stay (days)^e^	8 (6–13)	8 (5–14)	8 (6–13)	1.01 (0.95–1.07)

Data are presented as n (%) or median (interquartile range)

*BP* blood pressure, *CI* confidence interval, *ICH* intracerebral hemorrhage, *mRS* Modified Rankin Scale, *N/A* not available, *OR* odds ratio, *TOAST* Trial of ORG 10172 in Acute Stroke Treatment. ORs and 95% CIs were calculated via logistic regression

*Indicates statistical significance

^a^1 patient with recanalization and 43 patients with other treatments missing

^b^Pre-eclampsia, eclampsia, gestational hypertension, chronic hypertension and/or hemolysis, elevated liver enzymes, low platelets (HELLP)

^c^4 patients with other treatments missing

^d^22 patients with other treatments missing

^e^2 patients with other treatments missing

During the years 1995–2016 in Finland, there were 67 pregnant or postpartum IS patients, who were not given IVT. Thirteen patients had a reasoning in their patient records, explaining why they were not given IVT: two due to pregnancy, two due to a vaginal delivery within 2–3 weeks, and six due to a caesarean section within 2–4 weeks. However, one of these pregnant patients was treated with EVT alone. There were no physician notes regarding the exclusion of EVT (became available in 2015) due to pregnancy or a recent delivery.

## Discussion

In this nationwide study spanning 30 years, 12 of 97 (12.4%) pregnant or postpartum IS patients received recanalization therapy (8 IVT, 1 EVT, 3 combination therapy). Half of the patients received recanalization therapy during pregnancy and half in the postpartum period. IVT-treated maternal IS patients had a higher rate of early and major neurological improvements than the controls, and good functional outcomes at three months were equally common in both groups. Maternal IS patients treated with recanalization therapy had no other complications except one symptomatic ICH, and there were no perinatal deaths. Furthermore, when maternal IS patients with recanalization therapy were compared to those with conventional treatment, the occurrence of hemorrhagic transformation and mortality until the end of the follow-up time were similar. Good functional outcomes at the end of the follow-up time were five-fold more common if maternal IS patients had another treatment compared to recanalization therapy, possibly reflecting more severe strokes in patients receiving recanalization therapy.

Pregnant or postpartum patients treated with IVT were younger and had a higher percentage of dyslipidemia and a history of IS compared to non-pregnant controls treated with IVT, but other baseline characteristics did not differ. Also, in other studies comparing maternal and other IS patients who received recanalization therapy, pregnant and postpartum patients were younger, but their other baseline characteristics were similar [6–8]. Maternal IS patients had wake-up strokes more frequently than the controls, and although not statistically significant, the median NIHSS score was higher prior to treatment (10 vs 5). In a U.S. study, strokes were more severe in pregnant/postpartum IS patients than the controls [8], whereas the severity was similar in a French study [7]. Early and major neurological improvements were eight- to nine-fold more common in maternal IS patients than the controls. This might be partly explained by more severe strokes in pregnant/postpartum patients at the baseline, leading to a greater decrease in the NIHSS score.

The rate of any ICH did not statistically differ between IVT-treated maternal IS patients and controls, but this result must be interpreted with caution due to a low number of patients; two maternal ISs (18.2%) and 12 controls (6.1%) had any ICH. In prior studies, the risk of ICH and systemic bleeding after recanalization therapy in pregnant or postpartum patients compared to other IS patients was similar [7, 8]. Furthermore, the occurrence of any ICH was lower (11% vs 24%) in maternal ISs than other ISs in a study covering mechanical thrombectomy, in which a third of patients had concomitant IVT [6]. There was one symptomatic ICH according to the NINDS criteria (8.3%) and none according to the ECASS III criteria in maternal IS patients. The U.S. study had similar results, since the proportion of symptomatic ICH in IVT-treated pregnant/postpartum patients was 7.5% by the NINDS criteria [8]. In our study, there were no other complications than ICH, and no mortalities in IVT-treated maternal IS patients, which is in line with other studies reporting no mortalities or systemic hemorrhagic complications [7, 8], and 2.5% of other complications [8]. Patients with IVT had no preterm deliveries or perinatal deaths, as was found in the French study, in which all patients had live babies [7]. Good functional outcomes at three months were similar in IVT-treated maternal IS patients and controls (82% vs 87%). Other previously reported short-term outcomes include similar proportions of those discharged home in maternal and other IS patients [7, 8], and longer lengths of hospital stays in maternal ISs [6, 8].

When comparing maternal IS patients treated with recanalization therapy versus other treatments, the functional outcomes at discharge and at three months, maternal and perinatal mortalities, proportion of patients discharged home, and lengths of hospital stays were similar. Good functional outcomes at the end of the follow-up period were less common (67% vs 89%) in those treated with recanalization therapy, which might be partly explained by a shorter follow-up time and/or more severe strokes in them. Recanalization therapy has been studied poorly in pregnant and postpartum IS patients, so clinicians might have been hesitant to use recanalization for those with milder symptoms. Total and partial anterior strokes were more commonly treated with recanalization therapy, and posterior strokes more commonly with other treatments. Total anterior strokes tend to cause a more severe disability than other stroke subtypes [22]. In two prior studies, there were more severe strokes in pregnant or postpartum IS patients treated with recanalization versus other treatments [6, 7].

Recanalization therapy was either considered or given for approximately a third of maternal ISs after 1995, when the use of IVT for strokes began in Finland [23]. The refusal of giving IVT due to pregnancy or a recent delivery was quite rare. In a Canadian survey study, 92% of clinicians were hesitant to give IVT to otherwise eligible pregnant stroke patients if they had not encountered the issue before [24]. A recent major surgery is a relative contraindication of IVT, so a caesarean section was the most common recorded reason for not administering the treatment. Another reason for denying recanalization therapy might include a long delay between the onset of symptoms and admission to the stroke center. Patients treated with recanalization therapy were more likely admitted first to the stroke center than those that were treated otherwise, which might reflect good practices of emergency medical services or patient counseling, or simply because recanalization therapy was more likely considered in stroke centers. Finally, up to a third of maternal IS patients had hypertensive disorders of pregnancy; uncontrolled hypertension, as well as pre-eclampsia/eclampsia-related systemic complications, may make them ineligible for the treatment.

There are limitations in this study. The cohort size is small due to the rare entity of maternal ISs and the small size of the Finnish population (5.6 million in 2022). This could lead to a Type 2 statistical error, i.e., false negative results. Also, the logistic regression model could not be analyzed for all variables due to the lack of cases in certain subgroups. The Finnish population is relatively homogenic and the healthcare system is heavily subsidized, potentially leading to the results not being applicable to other populations. Pregnancies that resulted in induced or spontaneous abortions before 22 weeks of gestation were not included in our study, which might lead to the underestimation of the number of strokes in early pregnancies. Since the controls were derived from the Helsinki Thrombolysis Registry, they were treated only at Helsinki University Hospital, while maternal IS patients were treated nationwide. However, acute strokes are treated according to national evidence-based guidelines; access to acute stroke care is relatively equal across different parts of Finland, even though long distances in northern parts of the country may limit the number of eligible patients for IVT. The mRS and NIHSS scores were estimated for patients with maternal IS retrospectively using patient records if they were not recorded by the clinician, while the scores for controls were estimated based on the interview. The NIHSS score could not be estimated retrospectively in most conventionally-treated maternal IS patients, so we were not able to examine their stroke severity by NIHSS at hospital admission. Also, we could not analyze the delays between symptom onset and hospital admission in them since most patient records lacked data on that. The benefit of EVT has been proven for the first time in 2015, and therefore, the implementation of routine EVT started at the end of our study period [25]. In the future, EVT will likely replace IVT in patients with large artery occlusion if available. The strengths of this study are its nationwide nature, long sampling period, and comprehensive collection of pregnant and postpartum IS patients through reviewing medical records. The diagnoses and all clinical data of pregnant/postpartum IS patients were verified from patient records except the perinatal mortality, which was collected from registers.

To conclude, the use of recanalization therapy was rare in maternal IS patients in Finland. After IVT, good functional outcomes at three months were similar between maternal IS patients and other IS patients and severe complications were not observed among maternal IS patients. All IVT-treated pregnant/postpartum patients delivered full-term live babies. Among maternal IS patients, good functional outcomes at the end of the follow-up period were more common in patients with another treatment than recanalization, but the maternal and perinatal mortalities, proportion of patients discharged home, and the length of hospital stay were similar. According to the findings from our study and other register-based studies, the safety and efficacy of recanalization therapy during pregnancy and the postpartum period seemed similar to other IS patients. Randomized controlled trials on pregnant women and an evidence-based treatment would be ethically hard to justify and perform due to the rarity of maternal ISs. Thus, multi-center international register-based studies on the subject are needed to develop evidence-based guidelines for clinicians to determine the optimal acute treatment for IS during pregnancy and the postpartum period.

## Data Availability

As the data collected for this study includes the potential identification of patients and sensitive patient information, the data cannot be shared in open data repositories. De-identified aggregated data that support the findings of this study can be made available to qualified investigators on a reasonable request to the corresponding author.
